# A novel tool to evaluate and quantify radiation pneumonitis: A retrospective analysis of correlation of dosimetric parameters with volume of pneumonia patch

**DOI:** 10.3389/fonc.2023.1130406

**Published:** 2023-03-13

**Authors:** Jing-Wen Huang, Yi-Hui Lin, Gee-Chen Chang, Jeremy J. W. Chen

**Affiliations:** ^1^ Department of Radiation Oncology, Taichung Veterans General Hospital, Taichung, Taiwan; ^2^ Institute of Biomedical Sciences, National Chung Hsing University, Taichung, Taiwan; ^3^ Faculty of Medicine, School of Medicine, National Yang-Ming University, Taipei, Taiwan; ^4^ Institute of Medicine, Chung Shan Medical University, Taichung, Taiwan; ^5^ School of Medicine, Chung Shan Medical University, Taichung, Taiwan

**Keywords:** radiation pneumonitis, NSCLC, DVH, radiotherapy, pneumonia patch

## Abstract

**Introduction:**

In lung cancer, radiation-induced lung injury (RILI) or radiation pneumonitis (RP) are major concerns after radiotherapy. We investigated the correlation between volumes of RP lesions and their RP grades after radiotherapy.

**Methods and materials:**

We retrospectively collected data from patients with non-small lung cancer that received curative doses to the thorax without undergoing chest radiotherapy before this treatment course. The post-treatment computed tomography (CT) image was used to register to the planning CT to evaluate the correlation between dosimetric parameters and volume of pneumonia patch by using deformable image registration.

**Results:**

From January 1, 2019, to December 30, 2020, 71 patients with non-small cell lung cancer with 169 sets of CT images met our criteria for evaluation. In all patient groups, we found the RPv max and RP grade max to be significant (p<0.001). Some parameters that were related to the dose-volume histogram (DVH) and RP were lung Vx (x=1-66 Gy, percentage of lung volume received ≥x Gy), and mean lung dose. Comparing these parameters of the DVH with RP grade max showed that the mean lung dose and lung V1–V31 were significantly correlated. The cut-off point for the occurrence of symptoms in all patient groups, the RPv max value, was 4.79%, while the area under the curve was 0.779. In the groups with grades 1 and 2 RP, the dose curve of 26 Gy covered ≥80% of RP lesions in >80% of patients. Patients who had radiotherapy in combination with chemotherapy had significantly shorter locoregional progression-free survival (p=0.049) than patients who received radiation therapy in combination with target therapy. Patients with RPv max >4.79% demonstrated better OS (p=0.082).

**Conclusion:**

The percentage of RP lesion volume to total lung volume is a good indicator for quantifying RP. RP lesions can be projected onto the original radiation therapy plan using coverage of the 26 Gy isodose line to determine whether the lesion is RILI.

## 1 Introduction

In recent years, the global incidence of lung cancer has gradually increased (1), and radiation therapy has become a very important role in the treatment of lung cancer. Radiation therapy, a local treatment, is used for intervention in stage I–III lesions that are inappropriate for surgery, postoperative N2 nodal stage (2), oligometastasis (3), polymetastasis (4), or local recurrence/progression (5). However, radiation therapy used in treating the lung areas, including the mediastinal lymph nodes, can cause some damage to normal lung tissue. We refer to this as radiation-induced lung injury (RILI), or, more commonly, radiation pneumonitis (RP). Acute RILI mostly occurs within 6 months after thoracic radiotherapy (6, 7). The management of RP differs from that of other common lung lesions, for example, infection pneumonitis.

Distinguishing between the post-radiotherapy lung lesion and others can be used to direct the clinical treatment and avoid patient exposure to needless antibiotics use. To identify RILI radiation, oncologists need to correlate the post-RT CT images to the dose distribution map-related simulation image.

Since the correlation between volumes of RP lesions and their RP grades after radiotherapy has not been studied before and based on the advancement of modern planning software systems, we hope that in addition to a previous correlation between dose-volume histogram (DVH) and RP grades, fusion post-radiotherapy computed tomography and CT simulation can be used to explore the correlation between RP lesions and RP grading.

## 2 Methods and materials

We retrospectively collected data from patients with non-small lung cancer who underwent curative doses to the thorax (lung tumor, mediastinum lymph nodes, et. al) without undergoing chest radiotherapy before this treatment course. Patients with other malignancies were excluded within five years before lung cancer or uncompleted planned radiotherapy. The CT sets were excluded if subsequent CT confirmed locoregional progression due to hard-to-distinguish lesions between progression and pneumonitis. Further, when sputum culture proved an infection, that was also excluded. This study was approved by the Institutional Review Board of Taichung Veterans General Hospital (approval no. CE22095B). Written informed consent for participation was not required in accordance with national legislation and institutional guidelines.

### 2.1 Treatment planning

The intensity modulated radiation therapy (IMRT), and volumetric modulated arc therapy (VMAT) planned to use multiple field technique that was delivered to each patient by a linear accelerator (Varian 21EX and Varian 21iX with Millennium MLC-120, Varian Oncology Systems, Palo Alto, CA, USA) using 6 MV photons. The planning design of the Varian Eclipse planning system (version 6.5) was based on the Acuros XB algorithm. The treatment planning system of Tomotherapy was the Accuray planning station (Hi-Art, version 5.1.7). The treatment dose was 2 Gy once daily, five days a week, and the total dose was ≥56 Gy. Gross tumor volume (GTV) included the primary lung tumor, regional lymph nodes, and/or spine. Clinical target volume (CTV) included GTV plus 5 mm margin. Planning target volume (PTV) included GTV plus 5–6 mm margin. PTV-H refers to the PTV region that was covered by the highest dose. If the treatment field included the lung and bone, the prescribed dose for the bone was 40 Gy in 20 fractions. All plans were optimized to cover 100% of the PTV by 95% of the prescribed dose while minimizing the doses to the organs at risk (OAR) as much as possible.

### 2.2 RP evaluation

The CT images after radiotherapy were taken at a 5-mm thickness from the clavicular head to the second lumbar (L2) spinal vertebra level to cover the entire thorax, while the intravenous contrast enhancement was performed if there was no contraindication.

**Figure 1:**
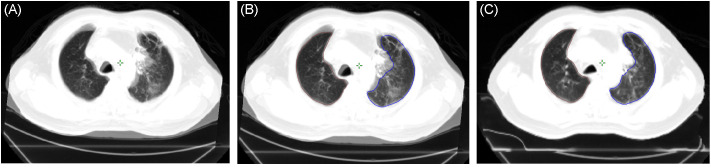
**(A)** Merged image of the post-radiotherapy CT and pre-treatment CT simulation based on a rigid registration process and showing significant inconsistency between respiratory status and lung volumes. **(B)** Lung volume of the pre-treatment CT simulation (blue: left lung, brown: right lung) projected on the post-radiotherapy CT (based on the rigid registration process). **(C)** Lung volume of the pre-treatment CT simulation projected on the post-radiotherapy CT (based on deformable image registration). CT, computed tomography.

We included the deformable image registration of registered CT images after radiotherapy in the CT simulation set. The deformable image registration (DIR) software was embedded in the Varian Eclipse planning system (8, 9). Plan of Tomotherapy (Tomo) was also imported into the Varian Eclipse planning system to calculate the overlapping prescribed dose and pneumonia patch. Because the CT breathing status at follow-up was not the same as that of the CT simulation, the pneumonia patches contoured on the CT during the follow-up period cannot correctly correspond to the image originally planned. Furthermore, inconsistencies in respiratory status can also lead to errors in volume percentages calculation and area of overlap assessment using the dose curve (**Figure 1**).

**Figure 2:**
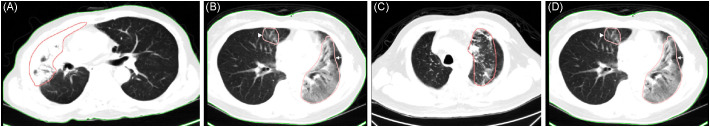
**(A)** Diffuse consolidation in a 70-year-old man with T4 N3 M0 non-small cell carcinoma, 70 days after completing radiotherapy (sequential chemotherapy followed by radiotherapy). **(B)** Diffuse ground-glass opacity (GGO) in a 55-year-old woman with T2a N2 M1c non-small cell carcinoma, 27 days after completing consolidative radiotherapy (arrow). **(C)** Patchy consolidation and GGO in a 75-year-old man with T4 N2 M0 non-small cell carcinoma, 98 days after completing concurrent chemoradiotherapy. **(D)** Patchy GGO same patient as **(B)** but occurring in the right lung (arrowhead).

The morphological evaluation of RP on CT appearance was judged according to Linda’s classification (10), while the labeling region included four patterns (1): diffuse consolidation (2), diffuse ground-glass opacity (GGO) (3), patchy consolidation and GGO, and (4) patchy GGO (**Figure 2**). Initial manual labeling of the CT scans was performed by a radiation oncologist (J-WH) with subsequent review by a thoracic medical doctor (G-CC). RP lesions on the grade 1 of CT images after radiotherapy were defined as RP1 (radiation pneumonitis grade 1), and so on, including the RP2 and RP3 and the calculated total volume of the original lung (based on the CT simulation). The volume ratio was defined as RPv1 (RP1 volume/total lung volume), RPv2 (RP2 volume/total lung volume), and RPv3 (RP3 volume/total lung volume). The severity of RP was assessed according to the Common Terminology Criteria for Adverse Events (CTCAE), ver.4.0. Furthermore, the grades of RP, when the CT images were evaluated, were defined as RP1, RP2, and RP3 grades, in chronological order. RP at follow-up for each patient was defined as RP grade max (radiation pneumonitis grade max), which was the most severe degree. Moreover, RP max volume/total lung volume was defined as RPv max. The time interval from completion of radiation therapy to RP grade max was defined as the number of days after completion of therapy until the onset of RP grade max or the onset of RPv max (for the same RP grade).

### 2.3 Survival analysis

Locoregional progression-free survival (LRPFS) was defined as the time from the completion of radiotherapy to the treatment field which received radiotherapy recurrence. Progression-free survival (PFS) was the time from the completion of radiotherapy to disease progression. Overall survival (OS) was the time from the completion of radiotherapy to death of any cause. Combined treatment, such as immunotherapy, will affect disease progression and survival. Thus, for analysis, LRPFS, PFS, and OS were collected by combining target therapy or chemotherapy groups.

### 2.4 Statistical analyses

Kruskal-Wallis test was used to determine if there were statistically significant differences between RP groups in independent variables, including DVH parameters and RPv max. Dunn-Bonferroni *post hoc* was used for pairwise comparison for the significant Kruskal-Wallis test. Mann-Whitney U test was used to compare the interval between grade 1 and grade ≥2 RP (symptomatic RP). The main application difference between the Mann-Whitney U and the Kruskal-Wallis H tests is that the latter can accommodate more than two groups (grade1, grade 2, and grade 3 pneumonitis). Spearman’s rank correlation coefficient was used to assess the relationship between RPv max (%) and lung Vx (x=1-66Gy, %). The log-rank test was applied to estimate LRPFS, PFS, and OS. The univariate Cox regression analysis was used to evaluate hazard ratio (HR) of prognostic factors on LRPFS, PFS, and OS. A p-value <0.05 was considered statistically significant. To predict symptomatic RP, the receiver operating characteristic (ROC) curve was used to compare the sensitivity versus specificity across a range of dosimetric parameters and RP volumes. All statistical analyses were performed by IBM SPSS Statistics for Windows, version 27 (IBM Corp., Armonk, NY, USA).

## 3 Results

From January 1, 2019, to December 30, 2020, 71 patients with non-small cell lung cancer met our criteria for evaluation, with a total of 169 sets of CT images, including 71, 55, and 43 sets of first CT images after receiving radiotherapy, 3 months after CT follow-up in the first group, and another 3 months afterward, respectively.

The patient characteristics are described in **Table 1**. The patients’ median age was 65 years (range, 42–86 years), the proportion of males was 60.56%, and the proportions of radiation therapy for curative, salvage, and consolidation purposes were 36.62%, 8.45%, and 54.93%, respectively. Doses ≥60 Gy accounted for 98.59% (n=70), while the lowest prescribed dose was 56 Gy. The dominant histological type was adenocarcinoma, accounting for 70%. The proportions of IMRT, VMAT, and Tomo in the treatment technology were 63.38%, 35.21%, and 1.41%, respectively. The combined treatment consisted mainly of target therapy and chemotherapy, with proportions of 49.30% and 35.21%, respectively. The treatment site was mainly the primary lung tumor, accounting for 56.34%. The proportions of RP grade max in grades 1, 2, and 3 were 51%, 45%, and 4%, respectively.

**Table 1:** Patient characteristics.

	Total (n=71)	
Age, years	65	(42–86)
Age ≥65 years	36	(50.70%)
Sex		
Female	28	(39.44%)
Male	43	(60.56%)
RT dose^1^(Gy)		
56	1	(1.41%)
60	49	(69.01%)
64	12	(16.90%)
66	9	(12.68%)
RT dose ≥60Gy	70	(98.59%)
Histology		
Adenocarcinoma	50	(70.42%)
Squamous	17	(23.94%)
Ca. PD	3	(4.23%)
Neuroendocrine	1	(1.41%)
Technique		
IMRT	45	(63.38%)
VMAT	25	(35.21%)
Tomo	1	(1.41%)
Treatment type		
No	8	(11.27%)
Target^2^	35	(49.30%)
C/T	25	(35.21%)
C/T + IO^3^	3	(4.23%)
Purpose		
Consolidative	39	(54.93%)
Curative	26	(36.62%)
Salvage	6	(8.45%)
Treatment site group		
Lung	40	(56.34%)
Lung+bone	3	(4.23%)
Lung+regional LNs	21	(29.58%)
Lung+regional LNs+bone	1	(1.41%)
Regional LNs	6	(8.45%)
PTV-H volume^4^(c.c)	199.20	(34.6-1020)
RP grade max^5^		
Grade 1	36	(51%)
Grade 2	32	(45%)
Grade 3	3	(4%)

^1^: highest prescribed dose.

^2^: Target therapy included tyrosine kinase inhibitor that targets EGFR and ALK, serine/threonine kinase (BRAF) inhibitor (dabrafenib) and mitogen-activated protein kinase (MEK) inhibitor (trametinib).

^3^: IO means immunotherapy.

^4^: PTV-H refers to the PTV region covered by the highest dose i.e., if the treatment field included the lung and bone, the prescribed dose for the lung was PTV-H and that for the bone was 40 Gy in 20 fractions.

^5^: RP grade max (radiation pneumonitis grade max) was defined as the most severe degree of RP at follow-up for each patient.

### 3.1 Interval to RP grade max

The first set of CTs was performed from 5 to 190 days; the interval to symptomatic RP grade max (grade ≥2) was from 24 to 190 days, to grade 3 RP was 27 to 70 days, and RP grade 0 was from 17 to 50 days (**Table 2**). If grade 3 RP occurs, it is usually distributed at ≤70 days after radiotherapy; on the contrary, for mild (grade <2) RP, especially grade 0 pneumonitis, intervals to RP grade 0 were from 17 to 50 days.

**Table 2:** RP grade max and interval of days to RP.

RP grade max^1^	Interval to RP1	Interval to RP2	Interval to RP3	Interval to RP max grade
1	44	133	233	133
1	80	164	269	80
2	30	136	216	136
2	126	231	301	126
2	105	217	329	105
2	120	218	407	120
1	36	127	304	127
2	72	184	296	72
1	112	210		112
2	135	248	357	135
1	17			17
1	111	195	279	279
1	5	102	201	201
1	131			131
2	190			190
1	135			135
2	84	168	252	84
1	25	117	229	117
1	75	141		75
1	50	217	302	302
2	34			34
2	51	161	273	51
2	119	203	315	119
1	55	149	239	149
1	43			43
1	35	133	231	133
1	55			55
1	64	161		64
2	24			24
2	46	136	227	136
2	60	144	215	60
1	24			24
2	45	150		45
3	27	128	212	27
2	62	178	311	62
1	17	114	224	114
2	85	268	357	85
1	55	167	293	55
1	23			23
2	83	181	265	83
2	124	208	293	124
2	128	240		128
2	21	103		103
1	112	224	315	112
1	93	177	317	93
3	48	154	287	48
2	71	147		71
1	83	195	251	83
1	28	126	245	28
2	76	139		76
1	25	130		25
2	36	148	218	148
1	139	230	323	139
2	64	169	295	64
2	98	182	276	98
2	106			106
1	75	131	243	243
1	65			65
2	110	229		110
1	98			98
1	41			41
1	17	80		80
3	70			70
1	73	185	241	185
2	52	89	206	89
1	29	134	260	134
1	96	195	308	96
2	88	172	284	88
2	69	181	281	181
1	31			31
2	98	203		98

^1^: RP grade max (radiation pneumonitis grade max) was defined as the most severe degree of RP at follow-up for each patient.

In the 169 CT series, 3 CTs without pneumonitis or consolidation lesion were found, also all that presented at first follow-up CT (in RP1 grade). Overall, all patients developed radiation-related lung injuries that could be detected by clinicians on post-radiotherapy CT.

### 3.2 Dosimetric parameters and volume of pneumonia patch

Symptomatic RP (grade ≥2) occurred more significantly in patients aged ≥65 years (p=0.006). Other factors such as sex, RT dose, or PTV-H volume did not differ between the two groups (**Table 3**).

**Table 3:** Patient characteristics in non-symptomatic and symptomatic RP.

	Symptomatic RP				p-value
	No (n=36)		Yes (n=35)		
Age, years	60	(42–86)	69	(50–79)	0.009**
Age ≥65 years	12	(33.33%)	24	(68.57%)	0.006**
Sex					0.735
Female	13	(36.11%)	15	(42.86%)	
Male	23	(63.89%)	20	(57.14%)	
RT dose^1^ (Gy)					0.107
56	0	(0%)	1	(2.86%)	
60	21	(58.33%)	28	(80.00%)	
64	8	(22.22%)	4	(11.43%)	
66	7	(19.44%)	2	(5.71%)	
Histology					0.257
Adenocarcinoma	25	(69.44%)	25	(71.43%)	
Squamous	8	(22.22%)	9	(25.71%)	
Ca. PD	3	(8.33%)	0	(0%)	
Neuroendocrine	0	(0%)	1	(2.86%)	
Technique					0.281
IMRT	25	(69.44%)	20	(57.14%)	
VMAT	10	(27.78%)	15	(42.86%)	
Tomo	1	(2.78%)	0	(0%)	
Combined treatment					0.247
No	3	(8.33%)	5	(14.29%)	
Target^2^	22	(61.11%)	13	(37.14%)	
C/T	10	(27.78%)	15	(42.86%)	
C/T + I/O^3^	1	(2.78%)	2	(5.71%)	
Purpose					0.660
Consolidative	20	(55.56%)	19	(54.29%)	
Curative	12	(33.33%)	14	(40.00%)	
Salvage	4	(11.11%)	2	(5.71%)	
Treatment site group					0.341
Lung	21	(58.33%)	19	(54.29%)	
Lung+bone	0	(0%)	3	(8.57%)	
Lung+regional LNs	12	(33.33%)	9	(25.71%)	
Lung+regional LNs+bone	0	(0%)	1	(2.86%)	
Regional LNs	3	(8.33%)	3	(8.57%)	
Treatment site group					0.939
Lung	21	(58.33%)	19	(54.29%)	
Lung+others	12	(33.33%)	13	(37.14%)	
Regional LNs	3	(8.33%)	3	(8.57%)	
PTV-H volume^4^ (c.c)	176.30	(34.6–1020)	211.20	(53.4–924.1)	0.337

Chi-Square test *p<0.05, **p<0.01.

Categorical variables are presented as frequencies and percentages (%). Continuous variables are presented as medians (ranges).

^1^: highest prescribed dose.

^2^: Target therapy including tyrosine kinase inhibitor that targets EGFR and ALK, serine/threonine kinase (BRAF) inhibitor (dabrafenib), and mitogen-activated protein kinase (MEK) inhibitor (trametinib).

^3^: IO means immunotherapy.

^4^: PTV-H refers to the PTV region covered by the highest dose i.e., if the treatment field included the lung and bone, the prescribed dose for the lung was PTV-H and that for the bone was 40 Gy in 20 fractions.

We compared RPv max with RP grade max in all patient groups and found a significant difference between RPv max and RP grade max (p<0.001). Some parameters related to DVH and RP included lung Vx (x=1-66 Gy, percentage of lung volume received ≥x Gy) and mean lung dose. Comparing the two parameters, DVH with RP grade max, showed that the mean lung dose and lung V1–V31 were significantly correlated (**Table 4**).

**Table 4:** RP grade max in all patient group.

	Lung Vx (x=1-66 Gy)												
	1 (n=36)			2 (n=32)			3 (n=3)			p-value	Dunn-bonferroni *post hoc*		
	Median		(IQR)	Median	(IQR)		Median	(IQR)			1 vs. 2	1 vs. 3	2 vs. 3
Mean lung dose (Gy)	10.0	(7.5-11.6)		10.7		(9-12.9)	17.8		(12.7-)	0.008**	0.202	0.015*	0.116
RPv max^1^ (%)	2.9%	(1.1%-5.5%)		6.6%		(3.9%-8.9%)	30.4%		(17.2%-)	<0.001**	0.001**	0.002**	0.122
1 Gy	74.1%	(51%-84.1%)		75.1%		(56.1%-88.9%)	94.7%		(89.8%-)	0.024*	1.000	0.020*	0.054
2 Gy	57.9%	(38.9%-64.6%)		59.1%		(39.7%-70.3%)	79.0%		(75.4%-)	0.023*	0.903	0.021*	0.071
3 Gy	49.3%	(33.7%-56.4%)		51.9%		(33.2%-59.6%)	68.7%		(65.5%-)	0.027*	0.902	0.025*	0.081
4 Gy	41.6%	(29.9%-52.1%)		46.6%		(30.9%-54.6%)	62.4%		(59.1%-)	0.033*	1.000	0.031*	0.090
5 Gy	38.0%	(26.2%-49%)		41.9%		(29.1%-49.7%)	57.5%		(54.2%-)	0.029*	1.000	0.026*	0.078
6 Gy	33.0%	(23.7%-45.7%)		36.6%		(26.1%-46.3%)	53.4%		(50%-)	0.023*	0.815	0.022*	0.079
7 Gy	29.7%	(21.7%-39.1%)		34.8%		(23.5%-42.4%)	49.8%		(45.8%-)	0.018*	0.593	0.020*	0.087
8 Gy	27.5%	(19.5%-36.5%)		32.0%		(22.5%-38.7%)	46.7%		(42.1%-)	0.019*	0.575	0.021*	0.094
9 Gy	25.7%	(18.3%-33.7%)		29.6%		(20.9%-36%)	43.9%		(39.3%-)	0.020*	0.557	0.023*	0.102
10 Gy	24.1%	(17.5%-31.6%)		27.6%		(20.4%-33.2%)	41.2%		(36.9%-)	0.015*	0.424	0.020*	0.103
11 Gy	22.8%	(16.5%-29.1%)		24.7%		(19.9%-31.8%)	38.6%		(34.5%-)	0.016*	0.464	0.020*	0.100
12 Gy	21.7%	(15.2%-26.7%)		23.5%		(19.3%-30.6%)	36.0%		(32.4%-)	0.019*	0.473	0.024*	0.115
13 Gy	20.4%	(14.9%-25.4%)		23.1%		(18.3%-29.4%)	33.5%		(30.5%-)	0.018*	0.409	0.025*	0.127
14 Gy	19.4%	(14.6%-23.6%)		21.9%		(17.7%-28.4%)	31.1%		(28.7%-)	0.017*	0.328	0.025*	0.144
15 Gy	18.3%	(14.3%-22.9%)		20.9%		(16.9%-27.6%)	29.0%		(27%-)	0.013*	0.217	0.025*	0.169
16 Gy	17.4%	(14%-22.2%)		19.9%		(16.1%-26.8%)	27.0%		(25.4%-)	0.012*	0.156	0.029*	0.219
17 Gy	16.5%	(13.7%-21.4%)		19.1%		(15.4%-26.1%)	25.2%		(24%-)	0.012*	0.130	0.035*	0.272
18 Gy	15.9%	(13.4%-20.9%)		18.4%		(14.7%-25.5%)	23.6%		(22.7%-)	0.013*	0.124	0.038*	0.291
19 Gy	15.3%	(13.2%-20.2%)		17.9%		(14.2%-24.9%)	22.0%		(21.6%-)	0.017*	0.113	0.059	0.413
20 Gy	14.8%	(12.9%-19%)		17.3%		(13.9%-23.8%)	20.6%		(20.6%-)	0.020*	0.097	0.089	0.577
21 Gy	14.3%	(12.5%-18.5%)		16.6%		(13.5%-22.7%)	19.8%		(19.3%-)	0.024*	0.097	0.117	0.700
22 Gy	13.7%	(11.8%-18.2%)		16.2%		(13.1%-21.8%)	19.1%		(18.1%-)	0.024*	0.090	0.124	0.741
23 Gy	13.3%	(11.3%-17.8%)		15.9%		(12.5%-20.2%)	18.4%		(16.9%-)	0.030*	0.117	0.130	0.714
24 Gy	12.9%	(11%-17%)		15.7%		(12.1%-19%)	17.7%		(15.9%-)	0.033*	0.117	0.149	0.783
25 Gy	12.4%	(10.7%-16.2%)		15.5%		(11.8%-18.3%)	17.1%		(14.8%-)	0.035*	0.101	0.187	0.952
26 Gy	11.9%	(10.1%-15.6%)		15.2%		(11.5%-17.8%)	16.5%		(13.9%-)	0.031*	0.084	0.193	1.000
27 Gy	11.3%	(9.1%-15.2%)		14.5%		(11.1%-17.3%)	16.0%		(13%-)	0.030*	0.067	0.238	1.000
28 Gy	11.0%	(8.3%-14.9%)		13.9%		(10.9%-16.9%)	15.4%		(12.1%-)	0.036*	0.068	0.321	1.000
29 Gy	10.7%	(8.1%-14.6%)		13.6%		(10.8%-16.6%)	14.9%		(11.2%-)	0.040*	0.065	0.399	1.000
30 Gy	10.5%	(7.8%-14.4%)		13.3%		(10.6%-16.3%)	14.4%		(10.3%-)	0.043*	0.060	0.514	1.000
31 Gy	10.2%	(7.6%-14.1%)		13.0%		(10.3%-15.5%)	13.9%		(9.3%-0%)	0.046*	0.054	0.682	1.000
32 Gy	9.7%	(7.3%-13.9%)		12.7%		(9.9%-14.8%)	13.3%		(8.3%-)	0.057			
33 Gy	9.3%	(7%-13.5%)		12.3%		(9.3%-14.3%)	12.7%		(7.3%-)	0.069			
34 Gy	9.0%	(6.7%-12.9%)		11.9%		(8.7%-14%)	12.2%		(6.3%-)	0.073			
35 Gy	8.7%	(6.4%-12.3%)		11.3%		(8.2%-13.8%)	11.6%		(5.4%-)	0.086			
36 Gy	8.4%	(6.2%-11.8%)		10.8%		(7.9%-13.5%)	11.0%		(4.5%-)	0.098			
37 Gy	8.2%	(6%-11.4%)		10.5%		(7.7%-13.3%)	10.6%		(3.5%-)	0.092			
38 Gy	7.9%	(5.8%-11.1%)		10.1%		(7.4%-13%)	10.2%		(2.3%-)	0.109			
39 Gy	7.5%	(5.6%-10.7%)		9.7%		(7.1%-12.7%)	9.8%		(1.2%-)	0.106			
40 Gy	7.2%	(5.4%-10.4%)		9.3%		(6.8%-12.2%)	9.4%		(0.4%-)	0.107			
41 Gy	6.9%	(5.2%-10%)		8.9%		(6.6%-11.7%)	9.0%		(0.1%-)	0.114			
42 Gy	6.7%	(5%-9.7%)		8.5%		(6.3%-11.2%)	8.6%		(0%-)	0.115			
43 Gy	6.4%	(4.8%-9.5%)		8.2%		(6.1%-10.8%)	8.3%		(0%-)	0.092			
44 Gy	6.1%	(4.6%-9.2%)		7.9%		(5.8%-10.5%)	7.9%		(0%-)	0.085			
45 Gy	5.9%	(4.4%-9%)		7.6%		(5.6%-10.2%)	7.6%		(0%-)	0.077			
46 Gy	5.6%	(4.2%-8.6%)		7.3%		(5.4%-9.9%)	7.2%		(0%-)	0.072			
47 Gy	5.3%	(4%-8.2%)		7.0%		(5.2%-9.5%)	6.8%		(0%-)	0.083			
48 Gy	5.1%	(3.7%-7.9%)		6.7%		(5%-9.2%)	6.4%		(0%-)	0.079			
49 Gy	4.9%	(3.5%-7.5%)		6.5%		(4.9%-8.8%)	6.0%		(0%-)	0.083			
50 Gy	4.7%	(3.3%-7.2%)		6.3%		(4.7%-8.5%)	5.6%		(0%-)	0.079			
51 Gy	4.5%	(3.2%-6.9%)		6.0%		(4.5%-8.2%)	5.3%		(0%-)	0.074			
52 Gy	4.2%	(3%-6.5%)		5.7%		(4.2%-7.8%)	4.9%		(0%-)	0.073			
53 Gy	4.0%	(2.8%-6.1%)		5.3%		(3.9%-7.5%)	4.6%		(0%-)	0.081			
54 Gy	3.8%	(2.5%-5.7%)		4.9%		(3.7%-7%)	4.3%		(0%-)	0.098			
55 Gy	3.4%	(2.4%-5.4%)		4.3%		(3.6%-6.5%)	4.0%		(0%-)	0.123			
56 Gy	3.2%	(2.2%-5%)		3.9%		(3.4%-6%)	3.6%		(0%-)	0.149			
57 Gy	3.0%	(2%-4.5%)		3.7%		(3%-5.7%)	3.2%		(0%-)	0.167			
58 Gy	2.8%	(1.7%-4%)		3.3%		(2.5%-5.2%)	2.9%		(0%-)	0.180			
59 Gy	2.5%	(1.6%-3.6%)		3.0%		(2%-4.6%)	2.4%		(0%-)	0.207			
60 Gy	2.2%	(1.4%-3.4%)		2.5%		(1.6%-4%)	2.0%		(0%-)	0.261			
61 Gy	1.9%	(1.2%-2.9%)		2.1%		(1.3%-3.5%)	1.5%		(0%-)	0.336			
62 Gy	1.6%	(1%-2.5%)		1.4%		(0.7%-2.7%)	0.9%		(0%-)	0.363			
63 Gy	1.2%	(0.5%-2.1%)		0.9%		(0.2%-2%)	0.5%		(0%-)	0.254			
64 Gy	0.9%	(0.2%-1.7%)		0.5%		(0%-1.4%)	0.3%		(0%-)	0.108			
65 Gy	0.6%	(0.1%-1.3%)		0.2%		(0%-0.6%)	0.1%		(0%-)	0.089			
66 Gy	0.4%	(0%-1%)		0.1%		(0%-0.4%)	0.0%		(0%-)	0.059			

Kruskal Wallis test. *p<0.05, **p<0.01.

^1^: the value of RP max volume/total lung volume.

When we differentiated between grades of RP with or without symptoms, there was also a significant difference in RPv max (p<0.001). Statistically significant differences in the lung mean dose, V10, V13–V46, and V48 were observed in the DVH portion of the lung (**Table 5**). Because age is also a factor in the occurrence of symptomatic RP, we divided the participants by age into ≥65 years and <65 years. Within these age groups, RPv max was significantly different between symptomatic and asymptomatic patients (**Table 6**), while mean lung dose only differed significantly between symptomatic and asymptomatic patients in the ≥65 years age group (p=0.041).

**Table 5:** Symptomatic RP.

	Lung Vx (x=1-66 Gy)				
	No symptoms (n=36)		Symptomatic (n=35)		p-value
	Median	IQR	Median	IQR	
Mean lung dose (Gy)	10.0	(7.5-11.6)	11.0	(9.4-14.5)	0.020*
RPv max^1^ (%)	2.9%	(1.1%-5.5%)	6.9%	(5%-10%)	<0.001**
1 Gy	74.1%	(51%-84.1%)	76.1%	(56.2%-89.8%)	0.175
2 Gy	57.9%	(38.9%-64.6%)	63.9%	(40.7%-71.5%)	0.121
3 Gy	49.3%	(33.7%-56.4%)	55.0%	(34.8%-64.7%)	0.123
4 Gy	41.6%	(29.9%-52.1%)	49.6%	(31.5%-59.1%)	0.144
5 Gy	38.0%	(26.2%-49%)	44.6%	(29.6%-54.2%)	0.141
6 Gy	33.0%	(23.7%-45.7%)	41.9%	(26.7%-50%)	0.107
7 Gy	29.7%	(21.7%-39.1%)	36.0%	(23.6%-45.3%)	0.073
8 Gy	27.5%	(19.5%-36.5%)	34.1%	(22.5%-41.5%)	0.071
9 Gy	25.7%	(18.3%-33.7%)	32.2%	(21.3%-39.3%)	0.069
10 Gy	24.1%	(17.5%-31.6%)	29.2%	(20.6%-36.9%)	0.049*
11 Gy	22.8%	(16.5%-29.1%)	27.9%	(20%-34.5%)	0.055
12 Gy	21.7%	(15.2%-26.7%)	26.7%	(19.3%-32.4%)	0.058
13 Gy	20.4%	(14.9%-25.4%)	24.4%	(18.6%-30.5%)	0.049*
14 Gy	19.4%	(14.6%-23.6%)	22.9%	(18%-29.4%)	0.038*
15 Gy	18.3%	(14.3%-22.9%)	21.4%	(17.5%-28.8%)	0.024*
16 Gy	17.4%	(14%-22.2%)	20.8%	(17%-27.3%)	0.017*
17 Gy	16.5%	(13.7%-21.4%)	20.4%	(16.7%-26.7%)	0.015*
18 Gy	15.9%	(13.4%-20.9%)	19.3%	(16.2%-26.1%)	0.014*
19 Gy	15.3%	(13.2%-20.2%)	18.2%	(15.6%-25.6%)	0.014*
20 Gy	14.8%	(12.9%-19%)	17.8%	(14.8%-24.4%)	0.013*
21 Gy	14.3%	(12.5%-18.5%)	17.5%	(14%-23.2%)	0.014*
22 Gy	13.7%	(11.8%-18.2%)	17.3%	(13.2%-22.2%)	0.013*
23 Gy	13.3%	(11.3%-17.8%)	16.9%	(12.6%-20.4%)	0.018*
24 Gy	12.9%	(11%-17%)	15.9%	(12.3%-19.1%)	0.018*
25 Gy	12.4%	(10.7%-16.2%)	15.6%	(12.1%-18.5%)	0.017*
26 Gy	11.9%	(10.1%-15.6%)	15.2%	(11.9%-17.8%)	0.014*
27 Gy	11.3%	(9.1%-15.2%)	14.5%	(11.8%-17.3%)	0.012*
28 Gy	11.0%	(8.3%-14.9%)	14.2%	(11.6%-17%)	0.013*
29 Gy	10.7%	(8.1%-14.6%)	13.9%	(11.2%-16.8%)	0.013*
30 Gy	10.5%	(7.8%-14.4%)	13.6%	(10.4%-16.5%)	0.013*
31 Gy	10.2%	(7.6%-14.1%)	13.3%	(10.2%-15.7%)	0.013*
32 Gy	9.7%	(7.3%-13.9%)	12.8%	(9.8%-14.8%)	0.017*
33 Gy	9.3%	(7%-13.5%)	12.4%	(9.2%-14.4%)	0.021*
34 Gy	9.0%	(6.7%-12.9%)	11.9%	(8.6%-14.1%)	0.022*
35 Gy	8.7%	(6.4%-12.3%)	11.3%	(8.1%-13.8%)	0.027*
36 Gy	8.4%	(6.2%-11.8%)	10.8%	(7.8%-13.6%)	0.032*
37 Gy	8.2%	(6%-11.4%)	10.5%	(7.6%-13.4%)	0.031*
38 Gy	7.9%	(5.8%-11.1%)	10.1%	(7.4%-13.2%)	0.040*
39 Gy	7.5%	(5.6%-10.7%)	9.7%	(7.1%-12.8%)	0.040*
40 Gy	7.2%	(5.4%-10.4%)	9.4%	(6.8%-12.2%)	0.043*
41 Gy	6.9%	(5.2%-10%)	9.0%	(6.5%-11.4%)	0.045*
42 Gy	6.7%	(5%-9.7%)	8.5%	(6.3%-10.8%)	0.048*
43 Gy	6.4%	(4.8%-9.5%)	8.2%	(6%-10.2%)	0.041*
44 Gy	6.1%	(4.6%-9.2%)	7.9%	(5.8%-10%)	0.040*
45 Gy	5.9%	(4.4%-9%)	7.6%	(5.5%-9.8%)	0.037*
46 Gy	5.6%	(4.2%-8.6%)	7.2%	(5.3%-9.6%)	0.041*
47 Gy	5.3%	(4%-8.2%)	6.9%	(5.2%-9.3%)	0.051
48 Gy	5.1%	(3.7%-7.9%)	6.7%	(5%-9.1%)	0.049*
49 Gy	4.9%	(3.5%-7.5%)	6.4%	(4.8%-8.8%)	0.053
50 Gy	4.7%	(3.3%-7.2%)	6.2%	(4.6%-8.4%)	0.054
51 Gy	4.5%	(3.2%-6.9%)	5.9%	(4.4%-8%)	0.054
52 Gy	4.2%	(3%-6.5%)	5.5%	(4.2%-7.6%)	0.051
53 Gy	4.0%	(2.8%-6.1%)	5.1%	(3.9%-7.2%)	0.055
54 Gy	3.8%	(2.5%-5.7%)	4.7%	(3.7%-6.8%)	0.070
55 Gy	3.4%	(2.4%-5.4%)	4.2%	(3.5%-6.5%)	0.090
56 Gy	3.2%	(2.2%-5%)	3.8%	(3.4%-5.9%)	0.119
57 Gy	3.0%	(2%-4.5%)	3.4%	(3%-5.7%)	0.146
58 Gy	2.8%	(1.7%-4%)	3.0%	(2.5%-5.2%)	0.212
59 Gy	2.5%	(1.6%-3.6%)	2.8%	(2%-4.5%)	0.314
60 Gy	2.2%	(1.4%-3.4%)	2.4%	(1.5%-3.8%)	0.577
61 Gy	1.9%	(1.2%-2.9%)	1.9%	(1.3%-3.4%)	0.913
62 Gy	1.6%	(1%-2.5%)	1.4%	(0.7%-2.6%)	0.538
63 Gy	1.2%	(0.5%-2.1%)	0.8%	(0.2%-1.9%)	0.192
64 Gy	0.9%	(0.2%-1.7%)	0.4%	(0%-1.2%)	0.047*
65 Gy	0.6%	(0.1%-1.3%)	0.1%	(0%-0.6%)	0.031*
66 Gy	0.4%	(0%-1%)	0.0%	(0%-0.4%)	0.018*

Mann-Whitney test. *p<0.05, **p<0.01.

^1^: the value of RP max volume/total lung volume.

**Table 6:** Symptomatic RP in the <65 and ≥65 years age group.

	Age <65 years					Age ≥65 years				
	Symptomatic RP				p-value	Symptomatic RP				p-value
	No (n=24)		Yes (n=11)			No (n=12)		Yes (n=24)		
	Median	IQR	Median	IQR		median	IQR	Median	IQR	
Mean lung dose (Gy)	10.1	(8-12.1)	10.7	(9.4-11.9)	0.334	9.1	(6-11.5)	11.1	(9-14.9)	0.041*
RPv max^1^ (%)	3.1%	(1%-5.5%)	5.9%	(3.2%-10.4%)	0.025*	2.6%	(1.7%-5.7%)	7.0%	(5%-9.7%)	0.003**
1 Gy	72.6%	(53.6%-84.2%)	69.5%	(47%-89%)	0.930	78.5%	(43.5%-84.1%)	77.9%	(59.5%-90%)	0.199
2 Gy	58.4%	(42.5%-64.6%)	58.5%	(35.3%-70.7%)	0.930	52.8%	(33.4%-64.5%)	66.2%	(43.6%-74.8%)	0.090
3 Gy	51.4%	(37.3%-56.1%)	48.2%	(31.4%-58.2%)	0.820	42.6%	(29.4%-57.5%)	56.9%	(38.8%-67%)	0.097
4 Gy	42.8%	(33.1%-51.3%)	41.9%	(28.4%-51.8%)	0.875	37.8%	(26.5%-54.1%)	51.0%	(35.5%-61.9%)	0.128
5 Gy	39.4%	(30.3%-47.2%)	38.4%	(25%-48.1%)	0.930	32.8%	(23.7%-51.6%)	46.0%	(31.3%-56.1%)	0.097
6 Gy	33.4%	(25.2%-43.7%)	36.0%	(22.1%-44.3%)	1.000	28.8%	(20.2%-46.7%)	42.7%	(28.6%-51.7%)	0.084
7 Gy	29.7%	(22.8%-38.3%)	34.1%	(20.4%-40.8%)	0.903	26.5%	(16.6%-44.5%)	39.8%	(24.9%-47.7%)	0.097
8 Gy	27.8%	(21.5%-36%)	31.7%	(19.9%-37.8%)	0.740	24.5%	(15.1%-42.5%)	36.7%	(22.6%-45.1%)	0.104
9 Gy	25.7%	(20.6%-33.3%)	28.5%	(19.5%-35%)	0.793	22.8%	(14.4%-37.2%)	33.7%	(21.8%-42.1%)	0.078
10 Gy	24.1%	(19.7%-30.8%)	26.4%	(19.1%-32.1%)	0.636	21.5%	(13.9%-32.8%)	32.2%	(20.8%-39.4%)	0.062
11 Gy	22.8%	(19.2%-28.5%)	24.1%	(18.8%-29.3%)	0.713	20.5%	(13.2%-30.1%)	30.0%	(20%-37.3%)	0.057
12 Gy	21.7%	(18.5%-26.7%)	22.2%	(18.5%-26.8%)	0.687	19.1%	(12.8%-27.5%)	28.0%	(19.4%-35.3%)	0.053
13 Gy	20.7%	(17.7%-25.4%)	21.8%	(17.9%-25.7%)	0.713	17.8%	(11.7%-25%)	26.6%	(18.7%-33.5%)	0.041*
14 Gy	19.6%	(17%-24.3%)	21.5%	(16%-24.9%)	0.793	16.8%	(11.1%-23.2%)	25.3%	(18.1%-32.3%)	0.026*
15 Gy	18.7%	(16.3%-23.3%)	21.1%	(14.6%-24.1%)	0.687	16.0%	(10.5%-22%)	24.1%	(17.5%-31.3%)	0.020*
16 Gy	18.0%	(15.7%-22.5%)	20.1%	(14%-23.4%)	0.587	15.3%	(9.9%-21.4%)	23.0%	(17.1%-30.4%)	0.013*
17 Gy	17.4%	(15.2%-21.9%)	19.0%	(13.8%-22.6%)	0.587	14.6%	(9.3%-20.9%)	22.0%	(16.7%-29.2%)	0.011*
18 Gy	16.7%	(14.7%-21.4%)	18.0%	(13.5%-22%)	0.587	14.1%	(8.9%-20.4%)	21.0%	(16.3%-27.6%)	0.010*
19 Gy	16.1%	(14.2%-20.8%)	17.2%	(13.3%-21.4%)	0.563	13.6%	(8.7%-19.6%)	19.9%	(15.6%-26.2%)	0.009**
20 Gy	15.3%	(13.7%-20.2%)	16.6%	(13.1%-20.6%)	0.540	13.2%	(8.4%-18.4%)	19.2%	(14.9%-25.5%)	0.010*
21 Gy	14.6%	(13.5%-19.7%)	16.4%	(12.9%-19.8%)	0.517	12.8%	(8.2%-17.3%)	18.6%	(14.1%-24.6%)	0.010*
22 Gy	14.1%	(13.1%-19.3%)	16.2%	(12.7%-18.9%)	0.472	12.5%	(7.9%-16.3%)	18.2%	(13.3%-23.4%)	0.007**
23 Gy	13.6%	(12.7%-18.8%)	16.0%	(12.5%-18.3%)	0.472	12.1%	(7.7%-15.4%)	17.7%	(12.7%-22.5%)	0.009**
24 Gy	13.0%	(12.3%-18.4%)	15.8%	(12.3%-17.8%)	0.472	11.8%	(7.6%-14.5%)	17.1%	(12.3%-21.7%)	0.009**
25 Gy	12.7%	(11.7%-18%)	14.8%	(12.1%-17.2%)	0.430	11.5%	(7.4%-13.8%)	16.4%	(11.9%-21%)	0.009**
26 Gy	12.5%	(11.2%-17.6%)	13.9%	(11.9%-16.6%)	0.375	11.2%	(7.2%-13%)	15.5%	(11.6%-20.4%)	0.010*
27 Gy	12.3%	(10.7%-17.2%)	13.6%	(11.8%-16%)	0.299	10.9%	(7%-12.4%)	14.7%	(11.3%-19.8%)	0.011*
28 Gy	12.0%	(10.2%-16.7%)	13.4%	(11.6%-15.4%)	0.316	10.6%	(6.8%-12%)	14.5%	(10.9%-18.8%)	0.012*
29 Gy	11.6%	(9.6%-16.2%)	13.2%	(11.2%-14.9%)	0.299	10.2%	(6.7%-11.5%)	14.2%	(10.6%-18.1%)	0.012*
30 Gy	11.1%	(9.2%-15.7%)	12.9%	(10.4%-14.7%)	0.238	9.8%	(6.4%-11.1%)	13.9%	(10.1%-17.5%)	0.012*
31 Gy	10.6%	(8.7%-15.3%)	12.7%	(10.2%-14.5%)	0.252	9.5%	(6.2%-10.7%)	13.5%	(9.6%-16.9%)	0.012*
32 Gy	10.1%	(8.3%-14.8%)	12.5%	(10.1%-14.3%)	0.238	9.2%	(5.9%-10.3%)	13.1%	(9.2%-16.2%)	0.022*
33 Gy	9.7%	(7.9%-14.1%)	12.3%	(10%-14.1%)	0.238	8.8%	(5.6%-10%)	12.6%	(8.8%-15.6%)	0.029*
34 Gy	9.3%	(7.6%-13.6%)	11.8%	(9.8%-13.9%)	0.224	8.5%	(5.3%-9.8%)	12.0%	(8.4%-15.1%)	0.032*
35 Gy	8.9%	(7.2%-13.1%)	11.3%	(9.6%-13.8%)	0.224	8.2%	(4.8%-9.7%)	11.4%	(7.9%-14.6%)	0.032*
36 Gy	8.6%	(6.9%-12.7%)	10.8%	(9.4%-13.6%)	0.252	7.9%	(4.4%-9.5%)	10.9%	(7.5%-14.2%)	0.038*
37 Gy	8.2%	(6.6%-12.2%)	10.3%	(9.1%-13.4%)	0.252	7.6%	(4.3%-9.3%)	10.5%	(7.1%-13.7%)	0.038*
38 Gy	8.0%	(6.3%-11.8%)	9.9%	(8.8%-13.2%)	0.268	7.4%	(4.1%-9.1%)	10.1%	(6.9%-13.1%)	0.041*
39 Gy	7.7%	(6%-11.4%)	9.5%	(8.4%-13%)	0.252	7.1%	(4%-9%)	9.8%	(6.7%-12.7%)	0.038*
40 Gy	7.5%	(5.8%-10.9%)	9.2%	(8.1%-12.7%)	0.238	6.8%	(3.9%-8.8%)	9.4%	(6.4%-12.1%)	0.041*
41 Gy	7.3%	(5.6%-10.5%)	8.8%	(7.8%-12.5%)	0.224	6.6%	(3.8%-8.6%)	9.0%	(6.2%-11.4%)	0.049*
42 Gy	7.0%	(5.4%-10.1%)	8.5%	(7.5%-12.3%)	0.211	6.3%	(3.7%-8.5%)	8.6%	(6%-10.7%)	0.062
43 Gy	6.8%	(5.2%-9.8%)	8.2%	(7.2%-12%)	0.163	6.0%	(3.5%-8.3%)	8.2%	(5.8%-10.1%)	0.061
44 Gy	6.6%	(5%-9.4%)	7.9%	(6.9%-11.8%)	0.152	5.7%	(3.4%-8.1%)	7.9%	(5.6%-9.8%)	0.057
45 Gy	6.3%	(4.8%-9.1%)	7.6%	(6.6%-11.5%)	0.152	5.4%	(3.3%-7.9%)	7.6%	(5.4%-9.5%)	0.041*
46 Gy	6.1%	(4.5%-8.8%)	7.4%	(6.3%-11.2%)	0.154	5.2%	(3.2%-7.7%)	7.2%	(5.2%-9.1%)	0.035*
47 Gy	5.8%	(4.3%-8.4%)	7.1%	(6%-11%)	0.201	4.9%	(3.2%-7.5%)	6.8%	(5.1%-8.8%)	0.045*
48 Gy	5.6%	(4.1%-8%)	6.8%	(5.6%-10.7%)	0.188	4.6%	(3.1%-7.3%)	6.4%	(4.9%-8.4%)	0.057
49 Gy	5.3%	(3.9%-7.6%)	6.6%	(5.3%-10.4%)	0.188	4.4%	(3%-7%)	6.0%	(4.7%-8.1%)	0.062
50 Gy	5.0%	(3.8%-7.3%)	6.3%	(4.9%-10.2%)	0.176	4.1%	(2.9%-6.8%)	5.7%	(4.5%-7.7%)	0.072
51 Gy	4.7%	(3.6%-7%)	6.1%	(4.6%-9.9%)	0.165	3.8%	(2.8%-6.4%)	5.3%	(4.3%-7.2%)	0.067
52 Gy	4.5%	(3.3%-6.7%)	5.8%	(4.2%-9.6%)	0.134	3.5%	(2.7%-6%)	5.0%	(4.1%-6.8%)	0.078
53 Gy	4.3%	(3%-6.4%)	5.6%	(3.9%-9.3%)	0.125	3.2%	(2.6%-5.6%)	4.7%	(3.9%-6.5%)	0.078
54 Gy	4.1%	(2.7%-6%)	5.3%	(3.6%-9%)	0.144	3.0%	(2.4%-5.2%)	4.4%	(3.7%-6.2%)	0.097
55 Gy	3.8%	(2.5%-5.5%)	5.0%	(3.3%-8.7%)	0.201	2.7%	(2.2%-4.8%)	4.1%	(3.6%-5.9%)	0.084
56 Gy	3.5%	(2.3%-5.1%)	4.8%	(3.1%-8.4%)	0.213	2.6%	(2%-4.4%)	3.7%	(3.4%-5.6%)	0.097
57 Gy	3.3%	(2.1%-4.7%)	4.5%	(2.9%-8.1%)	0.241	2.3%	(1.9%-4%)	3.3%	(3%-5.1%)	0.120
58 Gy	3.0%	(1.9%-4.3%)	4.1%	(2.6%-7.1%)	0.256	2.1%	(1.7%-3.7%)	3.0%	(2.4%-4.5%)	0.156
59 Gy	2.8%	(1.7%-3.9%)	3.8%	(2.3%-6.2%)	0.271	2.0%	(1.5%-3.3%)	2.7%	(1.8%-4%)	0.224
60 Gy	2.6%	(1.5%-3.4%)	3.3%	(1.8%-5.4%)	0.356	1.8%	(1.4%-3%)	2.4%	(1.3%-3.6%)	0.436
61 Gy	2.1%	(1.3%-3.1%)	2.8%	(1.5%-4.6%)	0.414	1.6%	(1.1%-2.7%)	1.9%	(0.9%-2.7%)	0.908
62 Gy	1.8%	(1%-2.9%)	2.0%	(1.1%-3.9%)	0.569	1.5%	(0.8%-2.1%)	1.0%	(0.5%-2%)	0.608
63 Gy	1.3%	(0.7%-2.6%)	1.3%	(0.6%-3.1%)	0.668	1.2%	(0.3%-1.6%)	0.7%	(0.1%-1.5%)	0.166
64 Gy	1.0%	(0.4%-1.9%)	0.8%	(0.2%-2.1%)	0.799	0.9%	(0.1%-1.4%)	0.3%	(0%-0.9%)	0.067
65Gy	0.7%	(0.2%-1.7%)	0.5%	(0.1%-1.2%)	0.563	0.5%	(0.1%-1.2%)	0.1%	(0%-0.4%)	0.097
66 Gy	0.5%	(0%-1.3%)	0.2%	(0%-0.5%)	0.351	0.2%	(0%-1%)	0.0%	(0%-0.1%)	0.068

Mann-Whitney test. *p<0.05, **p<0.01.

^1^: the value of RP max volume/total lung volume.

**Figure 3:**
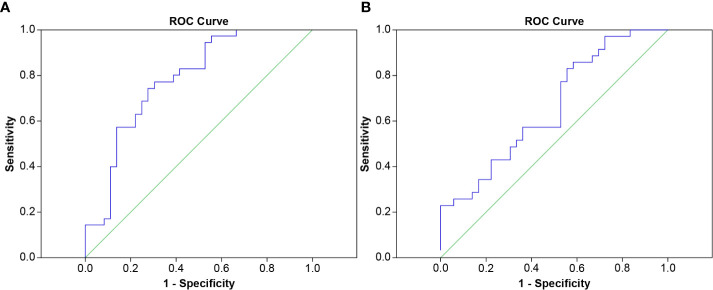
ROC curve of **(A)** RPv max **(B)** mean lung dose for predicting symptomatic RP.

The area under the ROC curve was used to evaluate the cut-off point for the occurrence of symptoms in all patient groups, the RPv max value was 4.79% while the mean lung dose was 8.4 Gy; the areas under the curve were 0.779 and 0.662 (**Figure 3**), respectively. Sensitivity, specificity, and the p values for the ROC curves of RPv max cutoff values were 0.77, 0.69, and <0.001, respectively. Sensitivity, specificity, and the p values for ROC curves of the mean lung dose cutoff values were 0.86, 0.42, and 0.02, respectively. Therefore, RPv max may have a better identification rate than the previous pulmonary dose curve distribution map for symptomatic RP.

### 3.3 Relationship between RP lesions and DVH

We found that V1-V12 and V25-V54 were significantly correlated to RPv max (**Supplementary Table S1**). The characteristic of the dose curve distribution is lower the dose, larger the coverage to the body, and vice versa. Therefore, in addition to the relationship between RPv max and lung Vx, when selecting a dose curve, we should use the curve with a higher dose as the principle. Finally, considering the comprehensive correlation and curve coverage to the largest patient population (>80% patients in grades 1 and 2 radiation pneumonitis, **Supplementary Table S2**) whose RP area is greater than 80%, V26 was selected.

**Figure 4:**
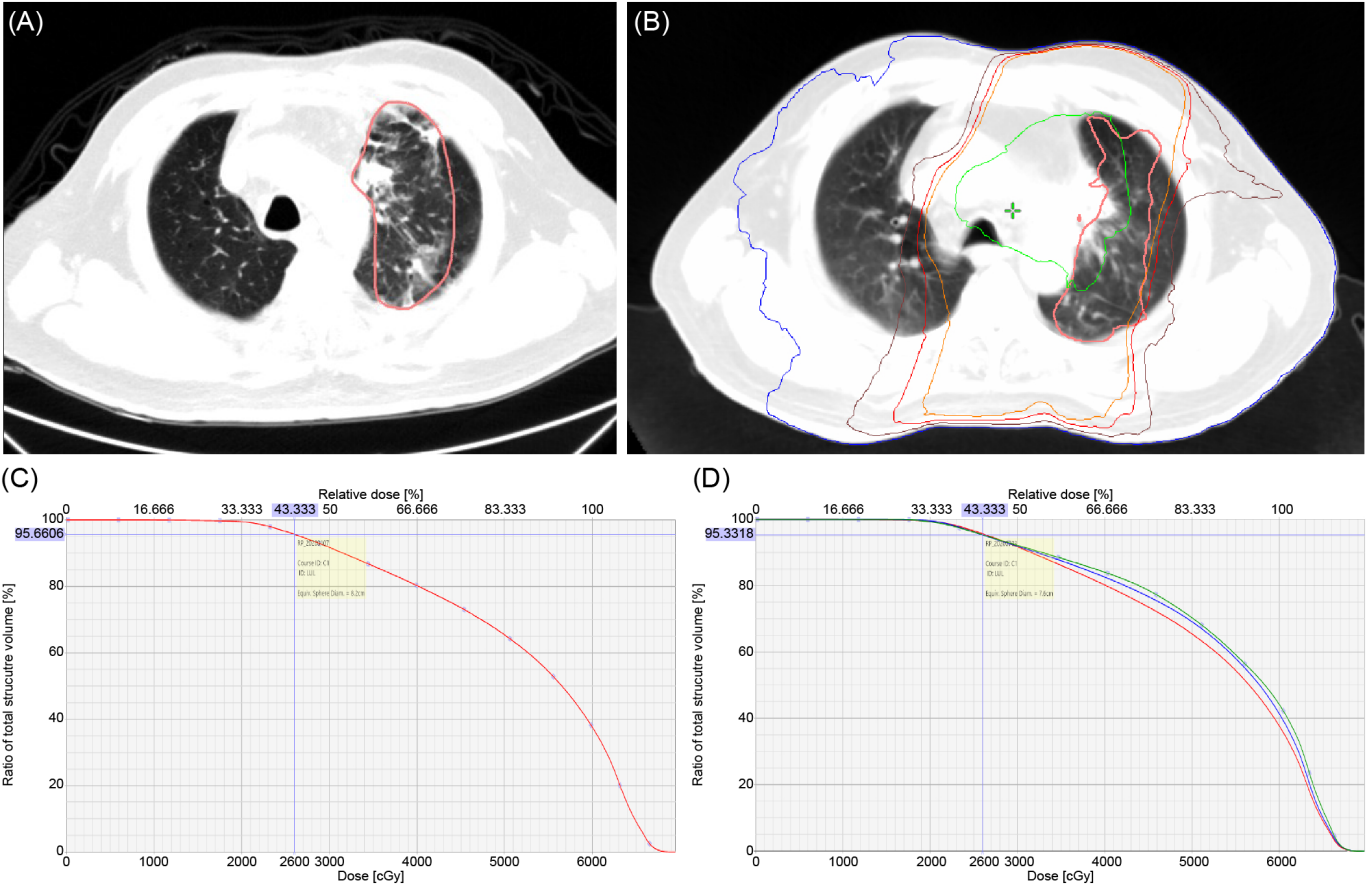
**(A)** RP lesion. **(B)** RP lesion covered by different isodose curve: blue – 5 Gy, brown – 20 Gy, red – 26 Gy, orange – 30 Gy, green – 60 Gy; pink – RP field projected to planning CT. **(C)** Demonstrated volume of RP 1 lesion covered by 26 Gy equal to 95.66%. **(D)** The percentages of RP1, 2, and 3 coverage drop rapidly when it exceeds 26 Gy (red: RP1, blue: RP2, green: RP3).

When we evaluated the correlation between RP lesions and DVH, in the grades 1 and 2 RP groups, the dose curve of 26Gy covered ≥80% of RP lesions (**Figure 4**) in the majority (80.6% and 81.3%, respectively) of patients (**Table 7**).

**Table 7:** Percentage of pneumonia patch covered by 26 Gy in grade 1 and 2 radiation pneumonitis patients.

Grade 1 (n=36)	Grade 2 (n=32)
99.9%	100.0%
99.4%	91.8%
99.5%	89.1%
100.0%	94.7%
99.5%	96.3%
100.0%	98.9%
100.0%	97.7%
99.6%	81.0%
89.9%	52.3%
100.0%	30.9%
86.0%	100.0%
100.0%	100.0%
58.0%	80.8%
72.8%	96.7%
98.5%	93.2%
100.0%	99.8%
99.2%	85.1%
99.8%	93.7%
96.3%	95.9%
69.8%	99.1%
77.4%	79.0%
100.0%	97.5%
99.5%	86.0%
100.0%	89.7%
95.0%	31.5%
79.6%	95.7%
87.6%	63.6%
100.0%	99.5%
98.4%	100.0%
98.2%	96.3%
59.2%	78.1%
89.8%	91.6%
98.6%	
99.9%	
0%	
100.0%	
≥80%, n=29	≥80%, n=26

Therefore, from the results presented in **Tables 4**–**6**, the ratio of RP lesion volume to the original lung volume after treatment was significantly correlated with the grade of RP; an RPv max value of 4.79% and mean lung dose of 8.4 Gy were the best indicators that were also significantly positively correlated with the RP grade.

### 3.4 Survival in radiotherapy combined target therapy or chemotherapy

**Figure 5:**
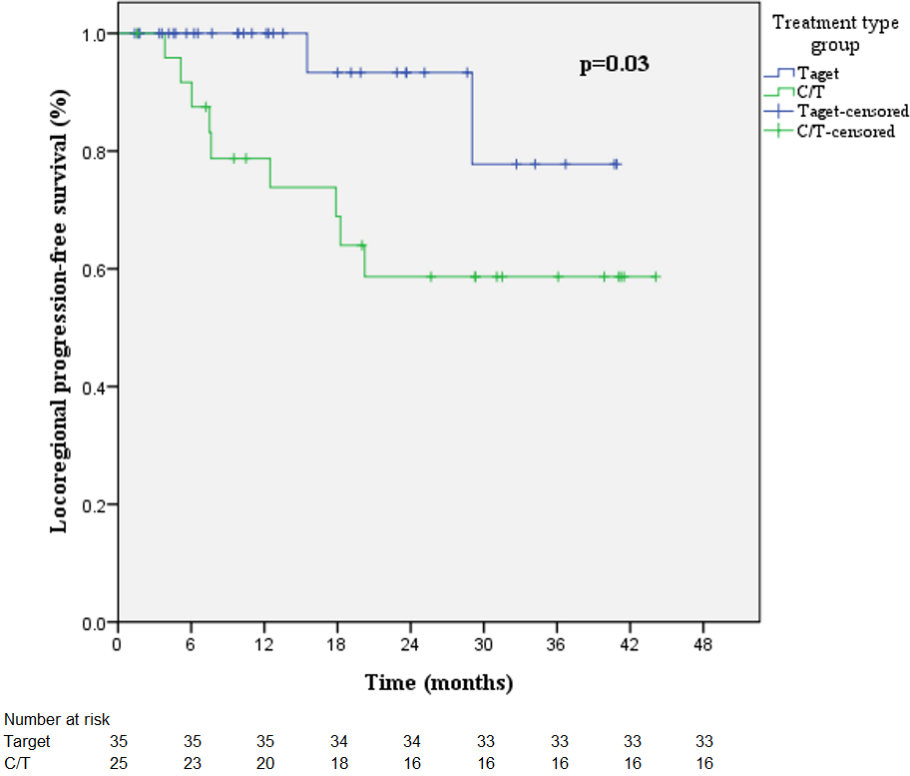
Kaplan-Meier curves of LRPFS in the radiotherapy-combined target therapy or chemotherapy treatment group.

The combining target therapy and chemotherapy groups were 35 patients and 25 patients, respectively. Percentage of mean lung dose >8.4Gy was significantly higher in combining chemotherapy than combining target therapy (p=0.01, **Supplementary Table S3**). However, symptomatic radiation pneumonitis was not different (p=0.08). Patients who received a combination of chemotherapy had significantly shorter LRPFS (HR 4.68 [95% CI 1.01-21.75], p=0.049), and the p value of log-rank test was 0.03 (**Supplementary Table S4**). Kaplan-Meier curves of LRPFS are presented in **Figure 5**. PFS and OS were not significantly different between combined target therapy and combined chemotherapy (**Supplementary Tables S5, 6**). Patients with RPv max >4.79% demonstrated a better trend of OS (HR 0.41 [95% CI 0.15-1.12], p=0.082) (**Supplementary Table S6**).

## 4 Discussion

We selected the curative dose in line with the current trend for definite concurrent chemoradiation therapy (CCRT) (60 Gy +/-10%) (11) because the use of definite RT for oligometastasis from the primary or metastatic site is considered when considering current radiation therapy for lung cancer. In terms of the patient characteristics, only age (≥65 years) affected the development of symptomatic RP (12). Others, such as PTV volume, were not associated with RP grade. This result is consistent with other studies’ results (13–15). Although these studies were based on high-dose radiation therapy, the dose to the normal lungs that actually affects the severity of RP was also revealed.

RP occurred within 6 months of radiotherapy completion. It manifests as radiation pulmonary fibrosis during the chronic phase (16–18). According to this study, symptomatic RP occurred in less than 190 days, similar to the results of Itonaga’s study (19).

A previous study has been conducted on the relationship between DVH and RP, although it is still debatable which is the best predictor of RP (20). One of the major reasons is that the assessment of RP has always been based on CTCAE, which is mainly graded according to the patient’s symptoms. Therefore, there is no objective way to quantify the degree of RP. In this study, by linking the percentage of RP lesion volume to the total lung volume and the degree of RP, we found that RPv max and RP did have a statistically significant relationship.

Palma et al. proposed changes in Hounsfield unit (HU) values on CT but found there was a poor correlation between HU density changes in the entire lung and the severity of physician-graded radiological pneumonitis (21). Two studies discovered that the HU density became denser between 3 and 9 months, then stabilized across all the dose intervals after 12 months (18, 22). The time distribution supports a RILI model with early RP and late fibrotic changes. The HU density changes in previous studies are similar to our observation of RPv max.

Several classification systems of RP were not clinically useful to allow for detailed, quantitative analysis of the radiological changes observed following RT or that have known correlations with clinical or dosimetric measures (10, 13, 23–25). Although Chandy et al. proposed five different RP types, their model presents sophisticated techniques for analyzing RP. Furthermore, it was impossible to classify them intuitively from the CT images (26). Szejniuk et al. reported the RILI grading scale (RGS), and dosimetric parameters were found to be related to changes in RGS severity (27). However, although RGS ranged from 0 to 3, the drawback of poor quantification is also noted. Itonaga et al. reported the range of pneumonia patches after stereotactic body radiation therapy (SBRT), and the median RILI ranges of the acute phase were in the 80% (20–100%) dose regions (19). Our study found that 26Gy can contain ≥80% of RP lesions in most patients. The implementation of SBRT has some clinical limitations for lung tumors that are located in the periphery and are <5 cm in size. Thus, the 80% dose regions were limited to the same lung as the treatment. However, the RILI ranges in conventional radiotherapy did not occur only in the same lung as that in Itonaga’s study. Moreover, only 3% of grade 2 RP appeared after SBRT and it is difficult to correlate the severity of RP with RILI ranges. Our study is innovative in the use of simple and objective data to quantify the severity of radiation pneumonitis. Furthermore, because there is no previous research on the relationship between the distribution of pneumonia and that of lung dose curves in conventional radiation doses, our data can provide a good way to identify radiation-induced lung injuries.

In combining target therapy or chemotherapy groups, patients who combined with chemotherapy had significantly shorter LRPFS than those combined with target therapy (p=0.049). In stage III-IV NSCLC, a prospective trial found that concomitant EGFR TKIs with RT produced positive results (28). In a preclinical model, radiation-induced antiangiogenic effects, anti-proliferation, and apoptosis were significantly enhanced in ALK-positive NSCLC cell lines while combined crizotinib (29). Moreover, a meta-analysis showed that local consolidative therapy (radiotherapy or surgery) with systemic treatment showed favorable results in both EGFR mutation and wild-type group than systemic treatment alone. HR for PFS in EGFR mutation population was 0.29 and in the wild-type population was 0.31 (30). Our study showed no difference in PFS or OS in combining target therapy or chemotherapy groups similar with the meta-analysis. Patients with RPv max >4.79% demonstrated a better trend of OS (p=0.082). After radiation, the immune system is strengthened, with tumor antigens being encouraged to stimulate the immune system and T cell detection and killing being strengthened (31). Wolf also found a reduced rate of disease failure, with disease failure occurring in 25.0% of individuals with radiographic radiation pneumonitis (RRP) and 80% of those without RRP (p=0.02) after stereotactic body radiation therapy (SBRT) (32).

The limitations of this study included the retrospective analysis. The time of the first CT after the completion of radiotherapy varied greatly; however, we could observe the difference in the degree of lung injury at different time points and processes. Another limitation was that different doctors might differ in the selection of the pneumonia lesions’ images. Therefore, in the future, we hope to use artificial intelligence (U-net) to achieve automatic segmentation by the computer, reduce subjective errors and predict the grade of lung injury. Also, by building automated programs to help clinicians, they can identify whether the lesions are lung injuries caused by radiation therapy. Even though 26Gy can contain ≥80% of RP lesions in most patients, a few patients still remained where the range of RP and those ≥26 Gy overlapped very little (or was even 0%). This means that radiation therapy to the lungs may also cause lung injury in low-dose areas. In this case, it is difficult to determine whether lung injuries were caused by radiation therapy or whether V26 covered the pneumonia patch. Therefore, other clinical manifestations (such as features of infection) are required to make a differential diagnosis. Since the purpose of treatment in our study includes definite concurrent chemoradiotherapy (curative), recurrence (salvage), and metastatic (consolidative) patients and varies combined treatment with radiotherapy, too many factors could affect survival. Thus, combining target therapy or chemotherapy groups was included for survival analysis. More patient numbers and longer follow-up time was needed to clarify the correlation between radiation pneumonitis and survival.

In conclusion, based on this study, we know that the ratio of RP lesion volume to total lung volume is a good indicator for quantifying radiation pneumonitis. The cut-off point for the occurrence of symptoms, the RPv max value, was 4.79%. RP lesions can be projected onto the original radiation therapy plan using coverage of the 26Gy isodose line to determine whether the lesion is RILI.

## Data availability statement

The original contributions presented in the study are included in the article/**Supplementary Material**. Further inquiries can be directed to the corresponding authors.

## Ethics statement

The human study was reviewed and approved by Taichung Veterans General Hospital (approval no. CE22095B).

## Author contributions

J-WH conceptualized the study. J-WH. and Y-HL collected the data. J-WH drafted the manuscript. G-CC and JC revised the manuscript and supervised the study. All authors read and approved the final manuscript. All authors contributed to the article and approved the submitted version.
