# Supplementary material for: A novel tool to evaluate and quantify radiation pneumonitis: A retrospective analysis of correlation of dosimetric parameters with volume of pneumonia patch
Source: Front Oncol. 2023 Mar 13;13:1130406. doi: 10.3389/fonc.2023.1130406 (PMC10040686; doi:10.3389/fonc.2023.1130406)
Supplement: Supplementary file 1 [file Table_1.docx]

**Supplementary Materials**

**Supplementary Table S1** Correlation coefficients (rs) between RPv max (%) and lung Vx (x=1-66 Gy, %)

|  | r_s_ | *p* value |
| --- | --- | --- |
| 1 Gy | 0.28 | 0.018* |
| 2 Gy | 0.24 | 0.044* |
| 3 Gy | 0.24 | 0.043* |
| 4 Gy | 0.25 | 0.039* |
| 5 Gy | 0.25 | 0.032* |
| 6 Gy | 0.29 | 0.016* |
| 7 Gy | 0.29 | 0.014* |
| 8 Gy | 0.28 | 0.020* |
| 9 Gy | 0.26 | 0.027* |
| 10 Gy | 0.26 | 0.030* |
| 11 Gy | 0.25 | 0.034* |
| 12 Gy | 0.24 | 0.044* |
| 13 Gy | 0.23 | 0.055 |
| 14 Gy | 0.22 | 0.063 |
| 15 Gy | 0.22 | 0.064 |
| 16 Gy | 0.21 | 0.073 |
| 17 Gy | 0.21 | 0.076 |
| 18 Gy | 0.22 | 0.070 |
| 19 Gy | 0.22 | 0.069 |
| 20 Gy | 0.22 | 0.071 |
| 21 Gy | 0.22 | 0.071 |
| 22 Gy | 0.21 | 0.077 |
| 23 Gy | 0.22 | 0.061 |
| 24 Gy | 0.23 | 0.057 |
| 25 Gy | 0.24 | 0.045* |
| 26 Gy | 0.26 | 0.031* |
| 27 Gy | 0.27 | 0.023* |
| 28 Gy | 0.28 | 0.019* |
| 29 Gy | 0.27 | 0.020* |
| 30 Gy | 0.28 | 0.019* |
| 31 Gy | 0.27 | 0.021* |
| 32 Gy | 0.27 | 0.022* |
| 33 Gy | 0.28 | 0.017* |
| 34 Gy | 0.29 | 0.015* |
| 35 Gy | 0.29 | 0.016* |
| 36 Gy | 0.28 | 0.018* |
| 37 Gy | 0.28 | 0.019* |
| 38 Gy | 0.28 | 0.019* |
| 39 Gy | 0.28 | 0.020* |
| 40 Gy | 0.27 | 0.023* |
| 41 Gy | 0.27 | 0.024* |
| 42 Gy | 0.27 | 0.025* |
| 43 Gy | 0.27 | 0.024* |
| 44 Gy | 0.26 | 0.028* |
| 45 Gy | 0.26 | 0.027* |
| 46 Gy | 0.26 | 0.031* |
| 47 Gy | 0.25 | 0.033* |
| 48 Gy | 0.26 | 0.031* |
| 49 Gy | 0.26 | 0.026* |
| 50 Gy | 0.26 | 0.029* |
| 51 Gy | 0.26 | 0.030* |
| 52 Gy | 0.26 | 0.030* |
| 53 Gy | 0.26 | 0.031* |
| 54 Gy | 0.24 | 0.041* |
| 55 Gy | 0.23 | 0.054 |
| 56 Gy | 0.21 | 0.082 |
| 57 Gy | 0.20 | 0.102 |
| 58 Gy | 0.17 | 0.150 |
| 59 Gy | 0.14 | 0.239 |
| 60 Gy | 0.09 | 0.433 |
| 61 Gy | 0.05 | 0.699 |
| 62 Gy | -0.03 | 0.804 |
| 63 Gy | -0.09 | 0.458 |
| 64 Gy | -0.14 | 0.230 |
| 65 Gy | -0.15 | 0.197 |
| 66 Gy | -0.19 | 0.114 |
| Spearman's rho. *p<0.05 | | |

**Supplementary Table S2** Percentage of patients with ≥80% pneumonia patches covered by 1–28 Gy in grades 1 and 2 radiation pneumonitis

|  | Grade 1 | Grade 2 |
| --- | --- | --- |
| 1 Gy | 97% | 100% |
| 2 Gy | 97% | 97% |
| 3 Gy | 97% | 97% |
| 4 Gy | 97% | 97% |
| 5 Gy | 97% | 94% |
| 6 Gy | 97% | 91% |
| 7 Gy | 97% | 88% |
| 8 Gy | 97% | 88% |
| 9 Gy | 92% | 88% |
| 10 Gy | 89% | 88% |
| 11 Gy | 89% | 88% |
| 12 Gy | 89% | 88% |
| 13 Gy | 89% | 88% |
| 14 Gy | 89% | 88% |
| 15 Gy | 89% | 88% |
| 16 Gy | 89% | 88% |
| 17 Gy | 89% | 88% |
| 18 Gy | 89% | 88% |
| 19 Gy | 86% | 88% |
| 20 Gy | 86% | 88% |
| 21 Gy | 83% | 88% |
| 22 Gy | 83% | 88% |
| 23 Gy | 83% | 88% |
| 24 Gy | 83% | 88% |
| 25 Gy | 83% | 84% |
| 26 Gy | 81% | 81% |
| 27 Gy | 81% | 75% |
| 28 Gy | 78% | 75% |

**Supplementary Table S3** Patient characteristics in the radiotherapy-combined target therapy or chemotherapy treatment group

| Combined treatment group | | | | | |
| --- | --- | --- | --- | --- | --- |
|  | Target (n=35) | | C/T (n=25) | | *p* value |
|  | n | (%) | n | (%) |  |
| Age, years (median, IQR) | 60.00 | (56-69) | 67.00 | (59-73.5) | 0.105 |
| Age ≧65 years | 13 | (37.14%) | 15 | (60.00%) | 0.080 |
| Sex |  |  |  |  | 0.011* |
| Female | 20 | (57.14%) | 6 | (24.00%) |  |
| Male | 15 | (42.86%) | 19 | (76.00%) |  |
| Purpose |  |  |  |  | <0.001** |
| Curative | 0 | (0%) | 18 | (72.00%) |  |
| Salvage | 2 | (5.71%) | 3 | (12.00%) |  |
| Consolidative | 33 | (94.29%) | 4 | (16.00%) |  |
| Mean lung dose (Gy) (median, IQR) | 8.96 | (7.53-10.65) | 11.57 | (9.99-14.85) | <0.001** |
| Mean lung dose (Gy)>8.4 | 20 | (57.14%) | 22 | (88.00%) | 0.010* |
| RPv max^1^ (%) (median, IQR) | 2.83 | (1.79-6.26) | 6.51 | (3.05-11.2) | 0.007** |
| RPv max (%) >4.79 | 15 | (42.86%) | 17 | (68.00%) | 0.054 |
| RP grade max^2^ |  |  |  |  | 0.137 |
| 1 | 22 | (62.86%) | 10 | (40.00%) |  |
| 2 | 13 | (37.14%) | 14 | (56.00%) |  |
| 3 | 0 | (0%) | 1 | (4.00%) |  |
| Symptomatic RP | 13 | (37.14%) | 15 | (60.00%) | 0.080 |
| Chi-Square test. **p*<0.05, ***p*<0.01. | | | | | |

^1^: RP max volume/total lung volume

^2^: RP grade max (radiation pneumonitis grade max) was defined as the most severe degree of RP at follow-up for each patient

**Supplementary Table S4** LRPFS in the radiotherapy-combined target therapy or chemotherapy treatment group

| LRPFS status | | | | |
| --- | --- | --- | --- | --- |
|  | Simple model | | | |
|  | HR | (95% CI) | | *p* value |
| Combined treatment |  |  |  |  |
| Target | 1.00 |  |  |  |
| C/T | 4.68 | (1.01- | 21.75) | 0.049* |
| Age≧65 years | 1.82 | (0.53- | 6.22) | 0.340 |
| Sex |  |  |  |  |
| Female | 1.00 |  |  |  |
| Male | 0.98 | (0.29- | 3.23) | 0.967 |
| Purpose |  |  |  |  |
| Curative | 1.00 |  |  |  |
| Salvage | 0.00 | (0.00- |  | 0.988 |
| Consolidative | 0.38 | (0.11- | 1.31) | 0.125 |
| Mean lung dose (Gy) | 1.15 | (0.95- | 1.38) | 0.144 |
| Mean lung dose (Gy) >8.4 | 3.26 | (0.42- | 25.53) | 0.260 |
| RPv max^1^ (%) | 0.98 | (0.90- | 1.06) | 0.581 |
| RPv max (%) >4.79 | 0.72 | (0.22- | 2.35) | 0.580 |
| RP grade max^2^ |  |  |  |  |
| 1 | 1.00 |  |  |  |
| 2 | 1.91 | (0.56- | 6.54) | 0.305 |
| 3 | 0.00 | (0.00- |  | 0.987 |
| Symptomatic RP | 1.78 | (0.52- | 6.10) | 0.359 |
| Cox regression. **p*<0.05, ***p*<0.01. | | | | |

^1^: RP max volume/total lung volume

^2^: RP grade max (radiation pneumonitis grade max) was defined as the most severe degree of RP at follow-up for each patient

**Supplementary Table S5** PFS in the radiotherapy-combined target therapy or chemotherapy treatment group

| PFS status | | | | |
| --- | --- | --- | --- | --- |
|  | Simple model | | | |
|  | HR | (95% CI) | | *p* value |
| Combined treatment |  |  |  |  |
| Target | 1.00 |  |  |  |
| C/T | 0.97 | (0.44- | 2.15) | 0.939 |
| Age≧65 years | 1.06 | (0.47- | 2.37) | 0.891 |
| Sex |  |  |  |  |
| Female | 1.00 |  |  |  |
| Male | 0.55 | (0.24- | 1.27) | 0.160 |
| Purpose |  |  |  |  |
| Curative | 1.00 |  |  |  |
| Salvage | 0.28 | (0.04- | 2.17) | 0.221 |
| Consolidative | 1.11 | (0.49- | 2.54) | 0.802 |
| Mean lung dose (Gy) | 0.98 | (0.86- | 1.11) | 0.715 |
| Mean lung dose (Gy) >8.4 | 0.59 | (0.25- | 1.36) | 0.214 |
| RPv max^1^ (%) | 0.97 | (0.92- | 1.03) | 0.308 |
| RPv max (%) >4.79 | 0.61 | (0.28- | 1.34) | 0.215 |
| RP grade max^2^ |  |  |  |  |
| 1 | 1.00 |  |  |  |
| 2 | 1.64 | (0.73- | 3.69) | 0.229 |
| 3 | 0.00 | (0.00- |  | 0.984 |
| Symptomatic RP | 1.50 | (0.67- | 3.36) | 0.328 |
| Cox regression. **p*<0.05, ***p*<0.01. | | | | |

^1^: RP max volume/total lung volume

^2^: RP grade max (radiation pneumonitis grade max) was defined as the most severe degree of RP at follow-up for each patient

**Supplementary Table S6** OS in the radiotherapy-combined target therapy or chemotherapy treatment group

| OS status | | | | |
| --- | --- | --- | --- | --- |
|  | Simple model | | | |
|  | HR | (95% CI) | | *p* value |
| Combined treatment |  |  |  |  |
| Target | 1.00 |  |  |  |
| C/T | 0.90 | (0.34- | 2.37) | 0.835 |
| Age≧65 years | 0.95 | (0.37- | 2.47) | 0.919 |
| Sex |  |  |  |  |
| Female | 1.00 |  |  |  |
| Male | 1.96 | (0.69- | 5.56) | 0.208 |
| Purpose |  |  |  |  |
| Curative | 1.00 |  |  |  |
| Salvage | 0.00 | (0.00- |  | 0.981 |
| Consolidative | 1.32 | (0.47- | 3.76) | 0.598 |
| Mean lung dose ( Gy) | 0.99 | (0.85- | 1.15) | 0.912 |
| Lung Mean ( Gy) >8.4 | 0.76 | (0.28- | 2.07) | 0.595 |
| RPv max^1^ (%) | 0.97 | (0.90- | 1.05) | 0.474 |
| RPv max (%) >4.79 | 0.41 | (0.15- | 1.12) | 0.082 |
| RP grade max^2^ |  |  |  |  |
| 1 | 1.00 |  |  |  |
| 2 | 0.56 | (0.21- | 1.53) | 0.257 |
| 3 | 0.00 | (0.00- |  | 0.983 |
| Symptomatic RP | 0.53 | (0.19- | 1.44) | 0.213 |
| Cox regression. **p*<0.05, ***p*<0.01. | | | | |

^1^: RP max volume/total lung volume

^2^: RP grade max (radiation pneumonitis grade max) was defined as the most severe degree of RP at follow-up for each patient
